# Genetic variations in the retrograde endocannabinoid signaling pathway in Chinese patients with major depressive disorder

**DOI:** 10.3389/fneur.2023.1153509

**Published:** 2023-04-20

**Authors:** Huifang Xu, Tongtong Li, Qiyong Gong, Haizhen Xu, Yongbo Hu, Wenqi Lü, Xin Yang, Jin Li, Wenming Xu, Weihong Kuang

**Affiliations:** ^1^Department of Psychiatry and National Clinical Research Center for Geriatrics, West China Hospital, Sichuan University, Chengdu, China; ^2^Department of Obstetrics and Gynecology, West China Second University Hospital, Sichuan University, Chengdu, China; ^3^Department of Obstetrics/Gynecology, Joint Laboratory of Reproductive Medicine (SCU-CUHK), Key Laboratory of Obstetric, Gynecologic and Pediatric Diseases and Birth Defects of Ministry of Education, West China Second University Hospital, Sichuan University, Chengdu, China; ^4^Department of Radiology, Huaxi MR Research Center (HMRRC), West China Hospital of Sichuan University, Chengdu, China

**Keywords:** major depressive disorder, whole exome sequencing, genetic mutation, retrograde endocannabinoid signaling, mitochondrial function

## Abstract

**Background:**

The retrograde endocannabinoid (eCB) pathway is closely associated with the etiology of major depressive disorder (MDD) at both pathophysiological and genetic levels. This study aimed to investigate the potential role of genetic mutations in the eCB pathway and underlying mechanisms in Han Chinese patients with MDD.

**Methods:**

A total of 96 drug-naïve patients with first-episode MDD and 62 healthy controls (HCs) were recruited. Whole-exome sequencing was performed to identify the gene mutation profiles in patients with MDD. Results were filtered to focus on low-frequency variants and rare mutations (minor allele frequencies <0.05) related to depressive phenotypes. Enrichment analyses were performed for 146 selected genes to examine the pathways in which the most significant enrichment occurred. A protein–protein interaction (PPI) network analysis was performed to explore the biological functions of the eCB pathway. Finally, based on current literature, a preliminary analysis was conducted to explore the effect of genetic mutations on the function of this pathway.

**Results:**

Our analysis identified 146 (15.02%) depression-related genetic mutations in patients with MDD when compared with HCs, and 37 of the mutations were enriched in the retrograde eCB signaling pathway. Seven hub genes in the eCB pathway were closely related to mitochondrial function, including Complex I genes (NDUFS4, NDUFV2, NDUFA2, NDUFA12, NDUFB11) and genes associated with protein (PARK7) and enzyme (DLD) function in the regulation of mitochondrial oxidative stress.

**Conclusion:**

These results indicate that genetic mutations in the retrograde eCB pathway represent potential etiological factors associated with the pathogenesis of MDD.

## 1. Introduction

Major depressive disorder (MDD) is a prevalent mental disorder that manifests as a wide spectrum of heterogeneous symptoms. As of 2019, MDD ranked 2nd among the top 25 leading causes of years lived with disability (YLDs) (1). However, the etiology and pathogenesis of MDD remain unclear. Therefore, understanding of the pathological mechanisms underlying MDD is necessary to promote effective treatment.

MDD is a complex disorder influenced by multiple genetic factors. The estimated heritability of MDD ranged from 30 to 50% (2). Although several primary genetic studies have focused on candidate genes that have been associated with MDD—including *SLC6A4, BDNF*, *COMT*, *HTR2A*, *TPH1* and *TPH2*—their results have provided little insight into the impact of these candidate genes on MDD (3). Several previous genome-wide association studies (GWAS) have identified common variations related to MDD. However, the estimated heritability of these common genetic mutations ranges from only 9 to 10% (4). Thus, researchers have shifted their attention to other undiscovered heritability, such as rare gene variations (MAF < 0.5%) and copy number variations (CNVs). Using whole-exome sequencing (WES), several studies have explored the contributions of rare or low-frequency variants to the genetic basis of MDD. One low-coverage whole-genome sequencing study identified the *SIRT1* and *LEPP* genes as risk loci in a sample of 5,303 Han Chinese women with recurrent MDD (5). The authors identified 1,985 variations in 479 MDD-related genes using different approaches and databases, reporting 14 gene mutations that differed between patients with MDD and the general South Asian population (6). A recent study that utilized 16,702 samples from the UK Biobank also highlighted the *FOXH1* gene and sphingolipid metabolism pathways as the most significant pathogenic genes for MDD (4). Despite several advances in understanding the genetic mutations underlying MDD, studies are still scarce and have limitations such as small sample sizes and large numbers of Europeans. Significant differences may exist across ethnicities due to the different allele frequencies. Therefore, it is necessary to study the genetic variation of Asians to expand the genetic research.

In the current study, we firstly explored the genetic variation profile of drug-naïve patients with MDD about whole-exome sequencing from China. Meanwhile, we conducted a genetic interaction analysis to infer functional variations *via* gene enrichment analysis rather than focusing on single-gene mutations. Preliminary explanation of how these genetic mutations affect physiological function were demonstrated through literature review.

## 2. Materials and methods

### 2.1. Participants and statistical analyses

A total of 96 first-episode drug-naïve patients with MDD and 62 healthy control (HC) participants were recruited for this study. All patients were recruited from the West China Hospital and had been diagnosed using the Structured Clinical Interview for DSM-IV Disorders (SCID). The inclusion criteria in this study were age of 18–60 years, presence of depressive symptoms for >2 weeks, and no previous exposure to antidepressant treatment. Exclusion criteria were as follows: history of psychosis, significant neurological or medical illness, current electroconvulsive therapy, and any history of alcohol or substance abuse or dependence. All HC participants, who were matched with the MDD group according to age and sex, were recruited from the local community through advertisements. Inclusion criteria for the HC group were as follows: no history of neuropsychiatric illness or brain injury, no family history of any serious mental illness in first-degree relatives.

Age was compared between the groups using nonparametric tests. Sex was compared using the chi-square test. The results are expressed as means ± SEM values and were analyzed by SPSS 26.0 (IBM, Chicago, IL, United States). The value of *p* < 0.05 is considered to be statistically significant.

### 2.2. Whole-genome sequencing and enrichment analyses

DNA samples were extracted and subjected to exome sequencing, including DNA quantification, library construction, exome sequencing, annotation and filtration. Detailed methods and data analysis are described in the Supplementary material.

To better predict the harmfulness of variation, we first utilized the classification system of the American College of Medical Genetics and Genomics (ACMG), which classifies variations as pathogenic, likely pathogenic, of uncertain significance, likely benign, or benign (7). Variations were then screened according to their scores using the SIFT (8), Polyphen (9), MutationTaster (10), and CADD (11) software programs. Potentially deleterious variations were retained if the scores from more than half of the four software programs supported their potential harmfulness (12).

Python scripts were used to extract SNVs and genes associated with a depressive phenotype (HP:0000716). Screening for variants with depressive phenotypes in patients and healthy controls revealed 146 variants that were specific to the patient group. We performed enrichment analyses on this set of 146 genes using clusterProfiler (13) and Metascape^1^ (14). Significant enrichment was defined as overlap of at least three genes, and the hypergeometric test was used to estimate the significance (*p* < 0.05). We also used Metascape to analyze the enrichment of mutated genes in the Kyoto Encyclopedia of Genes and Genomes (KEGG) pathway to further screen for these functions and interactions.

### 2.3. PPI network analysis

To identify the biological functions of the selected gene mutations in patients with MDD, 37 genes in the eCB pathway were mapped into the online search tool STRING database.^2^ A combined score of ≥0.4 was considered significant. Cytoscape software (^3^version 3.9.1; Institute for Systems Biology, Seattle, WA, United States) was used to construct and visualize the eCB gene variation network. To identify hub genes in the pathway, genes associated with other genes were ranked based on eigenvector centrality (EC) (15) using the CytoNCA v2.1.6 plugin. A survey of the current literature revealed that the top seven genes were related to mitochondrial function.

## 3. Results

### 3.1. Demographic and clinical characteristics

A total of 96 patients with MDD and 62 HCs were enrolled in this study. Age and sex did not significantly differ between the MDD and HC groups. The demographic and clinical information of the matched groups is presented in Table 1.

**Table 1:** Demographic and clinical characteristics of patients with MDD and healthy controls.

	MDD	HC	*p* value
Numbers (eCB/on-eCB)	96 (55/41)	62	
Age	31.829 ± 10.380	34.274 ± 10.090	0.129
Gender, M(W)	32 (64)	24 (38)	0.490
HAMD score	25.329 ± 8.166		

The value of *p* < 0.05 is considered to be statistically significant.

### 3.2. Characteristics of the genetic mutations and enrichment analysis

The 96 patients with MDD exhibited 15,637 SNPs of variant genes, 972 of which were related to the depressive phenotype. In the MDD group, we also extracted 3,353 SNVs involving 907 genes that were only related to depression phenotypes. Only a small number (146, 15.02%) of depression-associated gene mutations were observed among patients with MDD. The distributions of the 146 genes among patients with MDD are shown in Supplementary material.

**Figure 1:**
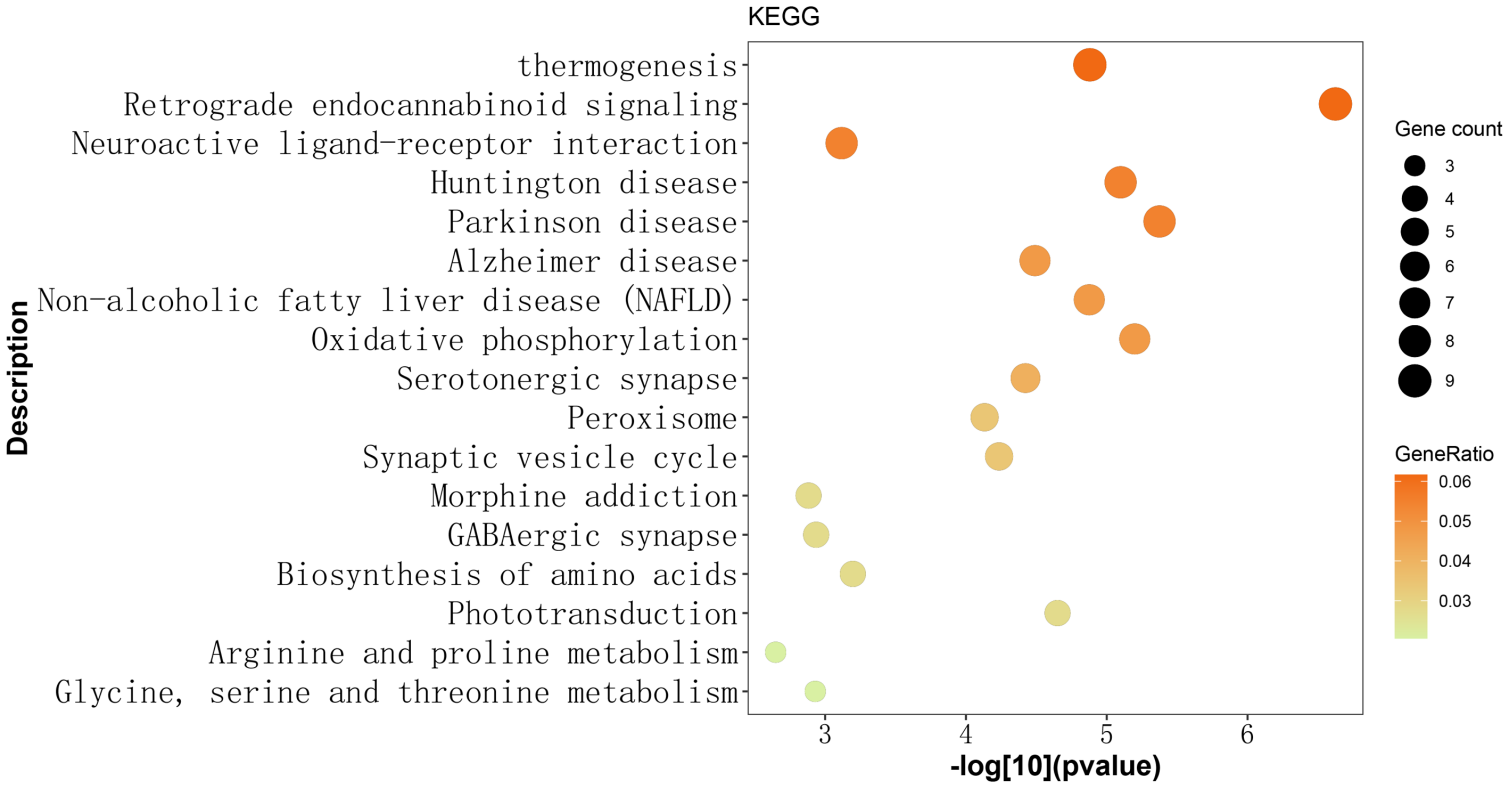
Identified 17 KEGG pathways based on 146 gene mutations observed exclusively in Chinese patients with MDD. The y-axis represents the KEGG pathway term, while the x-axis shows the -log[10](*p*-value). Each dot on the plot represents the fold enrichment for each KEGG pathway. The size of each dot corresponds to the number of mutated genes related to the depression phenotype, and the color of each dot represents the proportion of enriched genes in that pathway relative to the total number of mutated genes. The darker color indicates a higher proportion of genes. The retrograde endocannabinoid signaling pathway exhibited the most significant enrichment among the 17 KEGG pathways. KEGG: Kyoto Encyclopedia of Genes and Genomes.

Subsequently, enrichment analyses were performed for a set of 146 genes to investigate 17 pathways enriched in the KEGG pathway. Among these, the retrograde eCB signaling pathway was significantly associated with MDD (Gene count = 9, GeneRatio = 0.062, *p* = 0.000000238, -log10 (value of *p*) = 6.624). Additional details of 17 pathways were presented in Figure 1 and Supplementary material.

### 3.3. Genetic mutations in the retrograde eCB signaling pathway

**Figure 2:**
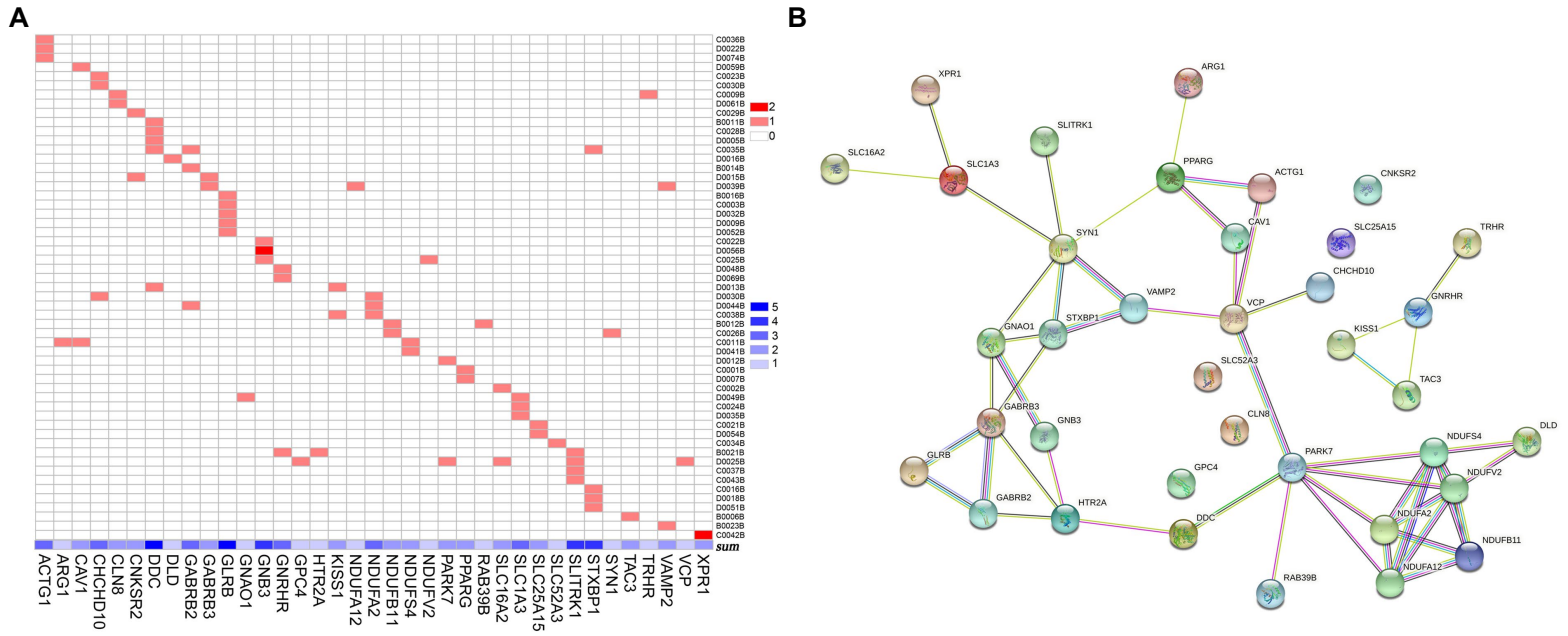
Gene mutations in the retrograde eCB signaling pathway in patients with MDD. **(A)** The 37 gene mutations in the retrograde eCB signaling pathway observed in 55 patients with MDD. The x-axis shows the 37 gene mutations, with the concentration of blue indicating the number of patients with MDD who carry each mutation. The y-axis shows each patient with MDD. The color in each square reflects the number of genetic mutations. Light red indicates that the gene is a heterozygous mutation, and dark red indicates a homozygous mutation. **(B)** The biological functions of the genes in the eCB signaling pathway based on the protein–protein interaction network analysis. Each node represents the proteins encoded by the genes, the contents of which are the 3D structures of proteins. Edges represent protein–protein associations contributing to a shared function. Different classifications are indicated by different colors. Known interactions are shown in blue and rose-red, based on information from curated databases and experimental studies, respectively. Predicted interactions are shown in green, red, and deep purple to indicate gene neighborhood, fusions, and co-occurrence, respectively. Yellow, black, and light purple represent interactions based on text mining, co-expression, and protein homology. eCB: endocannabinoid.

**Figure 3:**
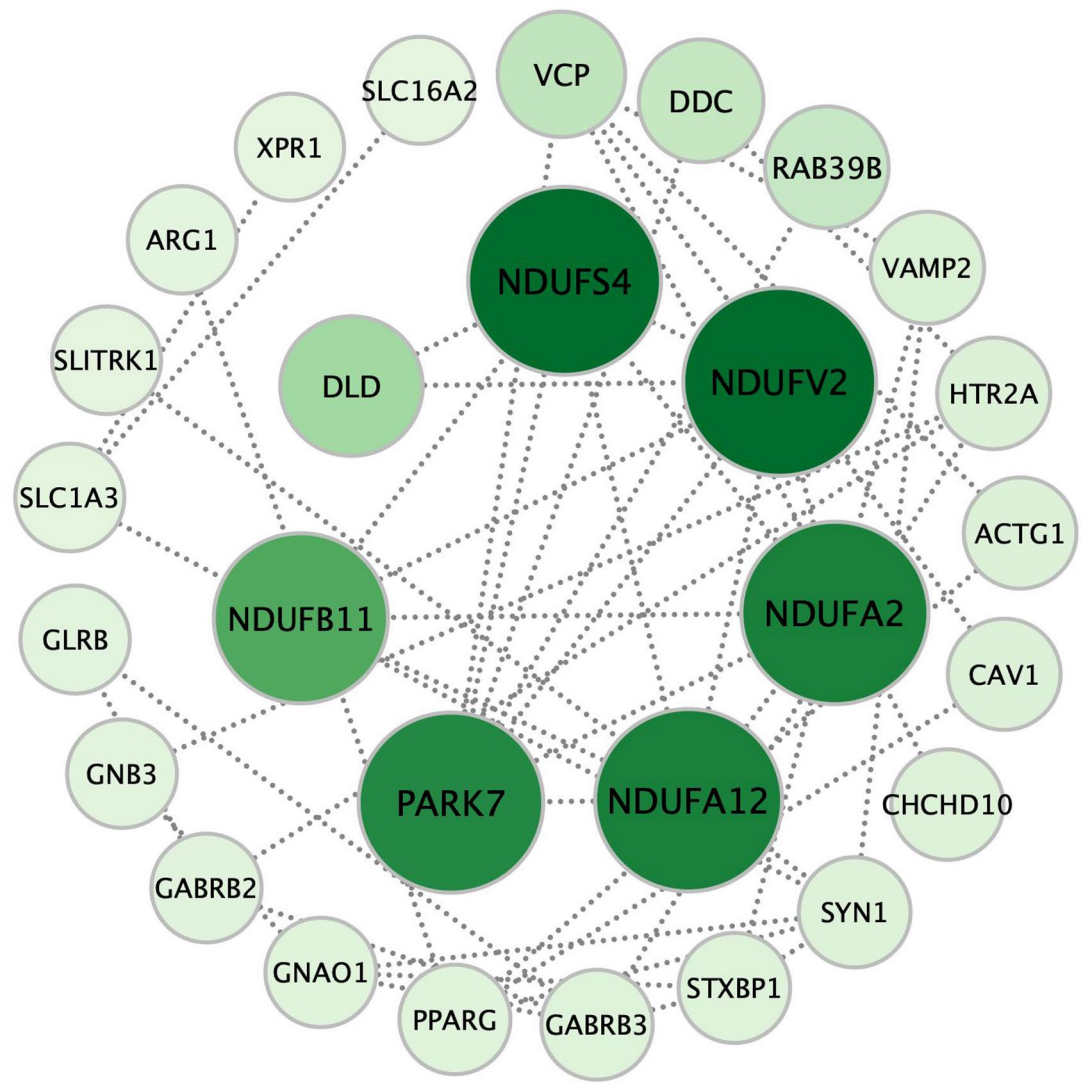
The seven hub genes of the retrograde eCB signaling pathway in patients with MDD based on the PPI analysis. Each circle represents a gene variation, and the sizes and color densities of each circle reflect ranking based on eigenvector centrality (EC). Larger, darker circles indicate the genes that are more significantly enriched in this pathway. The top seven most important genes are in the inner circle, while the others are arranged in the outer circle. eCB: endocannabinoid; PPI: protein–protein interaction.

There were 37 gene mutations in the retrograde eCB signaling in 55 patients with MDD (Figure 2). Among them, *DDC* and *GLRB* mutations were involved in the largest number of patients (five patients). Our PPI study demonstrated that seven hub genes were vitally related to mitochondria function, including Complex I (*NDUFS4*, *NDUFV2*, *NDUFA2*, *NDUFA12*, *NDUFB11*) and genes related to protein (*PARK7*) and enzyme (*DLD*) function in the eCB pathway, as shown in Figure 3. The details and frequency of the seven genes in the samples are presented in Table 2. In addition, *CHCH10* encodes a protein in the mitochondrial intermembrane that regulates mitochondrial function. Other gene variants included those related to synapse metabolism (*STXBP1* and *VAMP2*); protein-regulated neurotransmitters (*GNB3*, *GNAO1*, *STXBP1*, *SLC1A3*, *SLC16A2*); and receptors associated with glutamic acid (*GLRB*), gamma-aminobutyric acid (*GABRB2*, *GABRB3*), and 5-hydroxytryptamine (*HTR2A*). The 37 genes in the retrograde eCB signaling pathway were shown in Supplementary material.

**Table 2:** The seven hub genes mutated in eCB pathway in patients with MDD.

Gene	MDD	Chr	Cytoband	dbSNP	Ref	Alt	Effect	Clinvar	1KGP	ExAC	gnomAD	HUABIAO project:
NDUFS4	C0011	5	5q11.2	rs1064793807	GTG	CTC	Nonframeshift block substitution	Likely benign	NA	NA.	NA.	NA
	D0041	5	5q11.2	rs886060697	TTTG	-	splicing	Conflicting interpretations of pathogenicity	0.00139776	0.0032	0.003	NA
NDUFV2	C0025	18	18p11.22	rs769920941	G	C	Splicing	NA	NA	0.00001653	0.00001219	NA
NDUFA2	D0030	5	5q31.3	rs79526416	T	C	Missense	Uncertain significance	0.00079872	0.0001	0.0001	0.00232
	D0044	5	5q31.3	rs79526416	T	C	Missense	Uncertain significance	0.00079872	0.0001	0.0001	0.00232
	C0038	5	5q31.3	rs79526416	T	C	Missense	Uncertain significance	0.00079872	0.0001	0.0001	0.00232
NDUFA12	D0039	12	12q22	rs183579321	T	C	Missense	NA	0.00019968	0.00007421	0.00007716	0.00131
PARK7	D0012	1	1p36.23	rs756040385	G	A	splicing	Likely benign	NA	0.00004119	0.00003655	NA
	D0025	1	1p36.23	rs71653619	G	A	Missense	Benign	0.0061901	0.0081	0.008	0.0003
NDUFB11	B0012	X	Xp11.23	NA	G	C	Missense	NA	NA	NA	NA	NA
	C0026	X	Xp11.23	rs368074350	G	A	Missense	NA	NA	0.00002308	0.00002801	NA
DLD	D0016	7	7q31.1	rs200148324	C	T	Missense	NA	0.00039936	0.000008314	0.000008128	0.0005

Chr, chromosome; Ref, reference; Alt, allele; 1KGP, 1,000 Genomes Project; ExAC, Exome Aggregation Consortium; gnomAD, Genome Aggregation Database. NA, Not applicable.

## 4. Discussion

**Figure 4:**
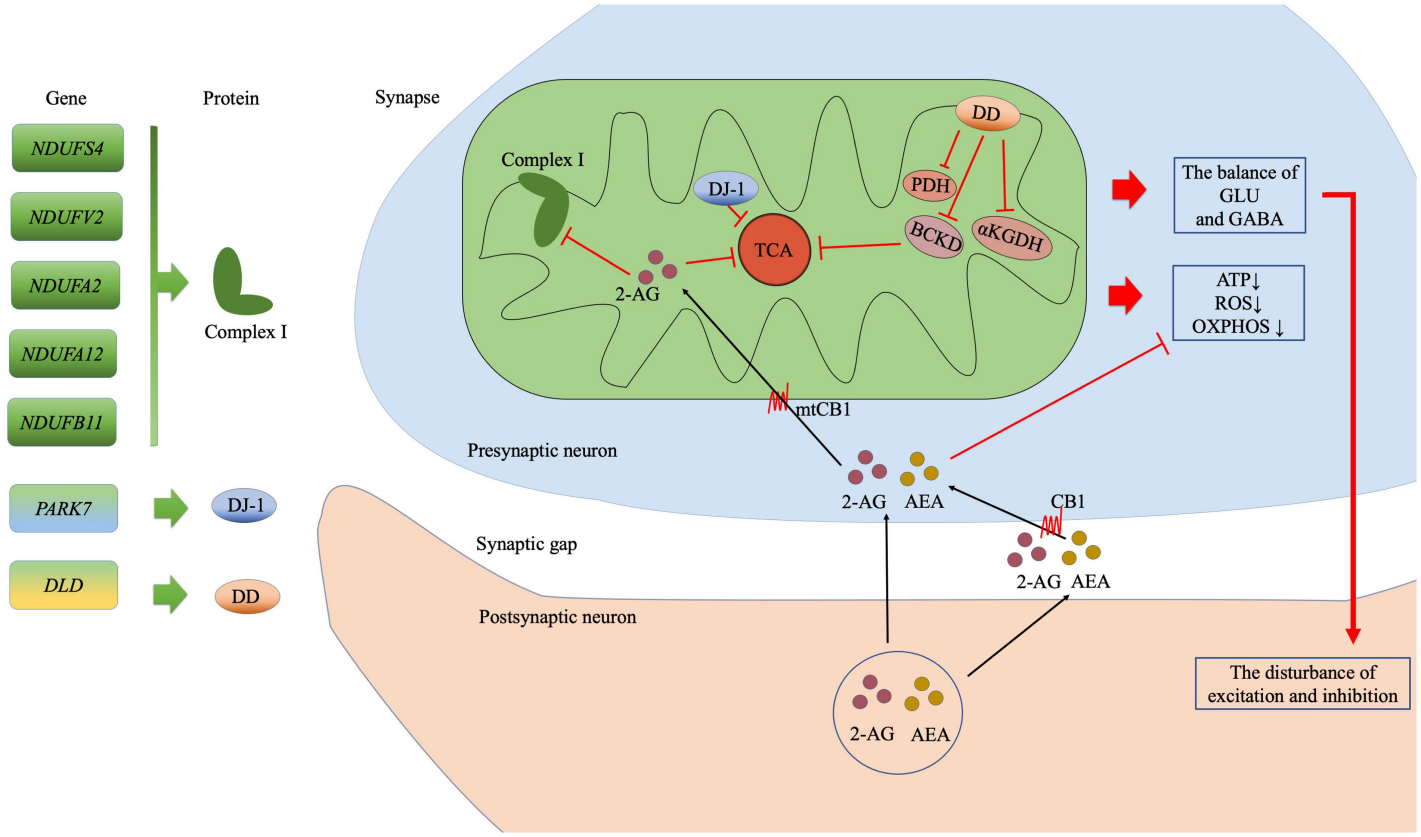
The possible mitochondrial mechanisms associated with the top seven genes in the eCB pathway analysis. The subunits of complex I are encoded by *NDUFS4*, *NDUFV2*, *NDUFA2*, *NDUFA12*, and *NDUFB11*. *PARK7* encodes the DJ-7 protein, while *DLD* encodes dihydrolipoamide dehydrogenase (DD), which in turn affects the enzyme complexes such as BCKD, PDH, and αKGDH. The eCBs 2-arachidonoylglycerol (2-AG) and anandamide (AEA) are synthesized in the postsynaptic membrane on demand. Then, 2-AG may enter the presynaptic membrane *via* CB or simple diffusion, following which it may inhibit oxidative function by acting on CB1 at the mitochondrial membrane (mtCB1) and regulating the subunits of complex I. Ultimately, the effects of this process on the TCA cycle reduced production of ATP, ROS generation, and reduced OXPHOS. In addition, disturbances in the balance of GLU and GABA exert an effect on synaptic function. Abbreviation: complex I: mitochondrial complex I (NADH: ubiquinone-oxidoreductase); BCKD: branched-chain alpha-keto acid dehydrogenase; PDH: pyruvate dehydrogenase; αKGDH: alpha-ketoglutarate dehydrogenase; TCA: tricarboxylic acid; ATP, adenosine triphosphate; ROS: reactive oxygen species; OXPHOS: oxidative phosphorylation; GLU: glutamate; GABA: γ-aminobutyric acid GABA; 2-AG: 2-Arachidonoylglycerol; AEA: anandamide or N-arachidonoylethanolamine; CB1: cannabinoid receptor 1; mtCB1: cannabinoid receptor 1 expressed on the mitochondrial membrane.

In the current study we found that 146 depression-associated genes were enrich 17 KEGG pathways and the retrograde eCB signaling pathway was the most notable. The 7 hub genes in this pathway were associated with the mitochondrial function in PPI. Our findings support the relationship between the eCB system and mitochondria in MDD from a genetic and biological function perspective. Figure 4 shows the possible underlying mechanistic pathways of the mutant gene-encoded proteins in mitochondria and the eCB signaling.

### 4.1. The close relationship between eCB system and major depressive disorder

We found that the retrograde eCB signaling pathway was significantly correlated with patients with MDD. The retrograde eCB signaling pathway, a component of the endocannabinoid system (ECS), is a widespread neuromodulatory pathway related to a range of physiological and pathological conditions, including the stress response, emotion, cognition, and memory (16). Numerous genetic and metabolomic studies have verified that abnormalities in eCB signaling play an essential role in MDD pathogenesis, impacting neurotransmission as well as the neuroendocrine and neuroimmune systems (17). Analyses based on the Psychiatric Genetic Consortium and UK Biobank have identified 43 differentially expressed genes between MDD and smoking in several neurotransmitter pathways, including the retrograde eCB signaling pathway (18). The retrograde eCB signaling was down-regulated pathway in bipolar disorder type I compared with depressive disorder based on expressed genes (19) and in bipolar II disorder (20). Another study detected 38 hippocampal metabolites related to retrograde eCB signaling in rats with prenatal stress that were associated with depression-like behaviors (21).

Furthermore, the polymorphisms of genes coding for the components of the ECS were related to MDD, such as cannabinoid receptors and the enzymes (22–25). Studies on enrichment analysis of genes involved in MDD also obtained that the neuroactive ligand receptor interaction (26), synaptic structure and neurotransmission (27), hypoxia, epithelial-mesenchymal transition, hedgehog signaling, and reactive oxygen species pathway (28) were achieved significance.

Our study complements the genetic mechanism of ECS in MDD as a biomarker and provides a theoretical basis for diagnosing and treating MDD. Activation of the ECS appears rapid-acting treatment for MDD (29). As conventional antidepressant drugs show delayed onset of therapeutic effects, novel treatments for MDD based on the ECS are developing rapidly. The endocannabinoid 2-Arachidonoylglycerol (2-AG) (17), the CB1 and CB2 receptors (30), and the enzyme (31) all have antidepressant pharmacological modulation and are potential new therapeutic targets for the treatment of MDD. Meanwhile, endocannabinoid-related compounds are also in rapid development. N-palmitoylethanolamide (PEA) is an endocannabinoid-like modulator, demonstrating an antidepressant-like effect (32). Cannabidiol, derived from phytocannabinoids, is a non-psychoactive substance that exerts antidepressant effects through multiple targets (33). ECS-based compounds are promising antidepressants in the future.

### 4.2. The biologically functional value of mitochondria in the eCB pathway

We identified 7 hub genes in the eCB signaling pathway, which were vitally related to mitochondria function. Among them, there are 5 genes encoding the mitochondrial Complex I (NADH: ubiquinone oxidoreductase), which is the largest inner membrane protein of the respiratory electron chain. The gene mutations in 45 subunits of Complex I have been closely linked to a wide range of neuropsychiatric disorders. *NDUFS4* (AQDQ protein) was identified as the most important site in the PPI of eCB in our cohort, as an accessory subunit associated with the assembly and/or stability of Complex I. The *NDUFS4* gene regulates the balance between excitatory (glutamate) and inhibitory (γ-aminobutyric acid, GABA) neurotransmission (34). An abnormal balance of glutamate and GABA is also usually found in patients with MDD. *NDUFV2* encodes a 24-kDa subunit of the NDUFV2 protein of Complex I. Initial studies have suggested a possible link between the mRNA level of *NDUFV2* and the state of bipolar disorder (BD) (35, 36). In addition, a haplotype T-C consisting of *NDUFV2* is most likely a protective factor for MDD in the Han Chinese population (37). *NDUFA2* encodes a subunit of the hydrophobic protein fraction of Complex 1. The mRNA level of *NDUFA2* was most significantly associated with schizophrenia (38) and the remission of psychiatric symptoms (39). *NDUFB11* (ESSS protein), located in the short arm of the X-chromosome, is essential for the assembly and activity of Complex I. *NDUFB11* may play a role in the mechanism underlying cognitive deficits in children and adolescents born preterm. Notably, cognitive impairment has been identified in more than half of patients with MDD (40). Although there have been no reports on *NDUFB11* and MDD, cognitive impairment may be a phenotype that this gene contributes to depressive disorder. *NDUFA12* is a small hydrophobic accessory subunit of Complex I, identified as a novel binding partner of the serine/threonine p21-activated kinase that increases susceptibility to type 2 diabetes (41). It is well-known that patients with MDD and diabetes are shared genetic risks (42). The underlying relationship between *NDUFA12* and MDD deserves to be explored. *PARK7* (DJ-7 protein) is a mitochondrial-associated protein, that exerts a wide range of effects on cellular functions including helping to prevent damage from reactive oxygen species (ROS), maintaining mitochondrial function, and participating in chaperone activity and carbohydrate metabolism (43). DJ-1 protein may act as an antioxidative defense mechanism to regulate mitochondrial dysfunction in the context of depressive disorders (44). *DLD* encodes a mitochondrial-associated enzyme called dihydrolipoamide dehydrogenase, which forms a subunit of several enzyme complexes, including pyruvate dehydrogenase (PDH) and α-ketoglutarate dehydrogenase (αKGDHc). Upregulation of *DLD* in the hippocampus was associated with anxiety-like behavior (45). The genes encoding mitochondria might affect depression-like behavior by regulating synaptic transmission, susceptibility, cognition and antioxidant.

The retrograde eCB signaling is involved in energy metabolism-regulated mitochondrial function *via* cannabinoid receptor 1 (CB1). CB1 is expressed not only on the cell membrane but also on the mitochondrial membrane (mtCB1) (46). The activity of mtCB1 exerts a great impact on brain mitochondrial physiology-associated bioenergetics, ROS production, and neurotransmitter regulation (47). Studies have indicated that mtCB1 can inhibit soluble adenylate cyclase and protein kinase A (PKA) activity, resulting in reduced PKA-dependent phosphorylation of mitochondrial proteins (46). Further, mtCB1 has been associated with the regulation of synaptic transmission, including glutamate and GABA transmission, *via* its effects on adenosine triphosphate supply and Ca^2+^ homeostasis (47). In addition, brain mtCB1 is important for regulating glutamate transmission associated with memory performance. The hippocampus is a vital regulator of memory and learning and is linked to acute mitochondrial activity in the brain. Hippocampal mtCB1 receptors regulate intra-mitochondrial Gαi proteins, resulting in the inhibition of soluble adenylyl cyclase (sAC), leading to a reduction in cAMP levels as well as decreased phosphorylation of PKA and subunit *NDUFS2* of Complex I. Eventually, this chain of activities regulates memory processes by decreasing the brain’s mitochondrial energy metabolism (48). Therefore, hippocampal mtCB1 is an important acute regulator of cognitive function. Astrocytes provide energy to the neurons in the brain by regulating cellular glucose metabolism. The activation of astroglial mtCB1 hampers glucose metabolism, reducing the generation of ROS and the phosphorylation of *NDUFS4* to destabilize Complex I, eventually impairing neuronal activity and behavioral responses in mice (49). The activation of muscular mtCB1, implicated in the metabolism of the primary tricarboxylic acid (TCA) substrate pyruvate, also participates in the regulation of oxidative activity (50).

Simply, mitochondrial-related genes lead to mitochondrial dysfunction affecting energy metabolism, oxidative stress, neurotransmitter transmission and cognitive function, mediated by mtCB1. Mitochondria have salient biological location in the eCB pathway.

### 4.3. The close relationship between mitochondria and major depressive disorder

Mitochondria are key organelles for energy production, involved in mechanisms of MDD through neuroimmune and neuroinflammation. The mobilization of energy is very important for dealing with stressful events. Mitochondria are involved in regulating the stress response mitochondrial biology is tissue- and cell-specific, particularly in the immune system (51). Social stress is a risk factor for the development of MDD (52) and contributes to mitochondrial dysfunction, leading to inflammatory disturbances (53). One study of metabolomic signatures reported that mitochondrial oxidative phosphorylation (OXPHOS), morphology, and recycling were crucial elements of the stress response. In addition, the authors reported upregulated protein expression of Complexes I, II, and IV in resilient animals, demonstrating that the electron respiratory chain is positively associated with chronic stress (52). Another study found that the changes in protein levels related to mitochondrial dysfunction were dependent on peripheral inflammation, which regulates the severity of MDD (54). Moreover, plasma levels of inflammatory cytokines such as C-reactive protein (54) and interleukin-6 (55) are regarded as markers of mitochondrial disturbance and are known to modulate the severity of depressive symptoms. A longitudinal study showed of patients with treatment-resistant depression undergoing anti-inflammatory treatment with the tumor necrosis factor antagonist infliximab identified peripheral blood gene transcripts enriched for oxidative stress and mitochondrial degradation, which were related to increases in psychomotor reaction time (55). The inflammatory signaling and metabolic reprogramming in the immune system were associated with inflammation in patients with depressive disorders and may promote psychomotor retardation. In another study, alterations in the dynamic regional homogeneity of the brain were observed between 65 first-episode, treatment-naïve patients with MDD and 66 HCs, which correlated with the 16 gene modules investigated in the weighted gene co-expression network analysis. The expression profiles of the gene modules were enriched for immune, mitochondrial, protein, and synaptic signaling (56).

Taken together, multiple lines of evidence indicate that stress regulation and neuroimmunology are key areas linking mitochondrial function and MDD etiology. Genetic studies may also provide novel insights into different antioxidants and anti-immunotherapies that are safe for adjunctive treatment of depressive symptoms (57). Based on the available evidence and the current findings, these therapies may be ideal for patients with MDD exhibiting stress-related immune impairments.

### 4.4. Limitation and further investigation

Although our findings provide insight into the role of eCB gene mutations in the pathogenesis of MDD, the current study was limited by its small sample size and the use of a literature review to examine potential molecular mechanisms. As this was a cross-sectional study comparing patients with MDD to HCs, additional studies are also required to determine the direction of causality. Moreover, future studies should aim to examine the impact of epigenetic modifications, the expression profile of these gene variants, and real gene–gene interactions in the context of MDD. Given that MDD is a multifactorial disorder influenced by genetic and environmental factors and the interactions between them, these elements should also be included in future studies. Lastly, we were unable to exclude potential confounders known to affect mitochondrial function, such as lifestyle factors, childhood trauma, chronic stress, and suicidal behaviors. The current study should be considered exploratory, and its findings must be verified in a large-scale longitudinal cohort and validated experimental models.

It is noteworthy that MDD is a complex multifactorial disease. Several mechanisms for MDD pathogenesis have been proposed including neuroinflammation, neurotransmitter abnormalities, neuroendocrine dysfunction, mitochondrial abnormalities, and altered stress regulation. No single factor impacts the final effect, as each factor can both modulate and is modulated by other factors, resulting in intricate relationships and complex interactions. The complex relationship between mitochondrial function and the manifestation of MDD remains unclear, and the current findings must be interpreted with caution.

## 5. Conclusion

In conclusion, our analysis identified profiles of genetic variation in Han Chinese patients with first-episode MDD and the possible genetic mechanisms by which the retrograde eCB pathway exerts an influence of MDD in these patients. Our preliminary study broadens the current understanding of the complex genetic basis of MDD and highlights genetic mutations in the eCB pathway as potential etiological factors associated with the pathogenesis of MDD. Identifying specific mutations in this pathway may be beneficial for targeted MDD therapy in the future.

## Data availability statement

The data presented in the study are deposited in the zenodo repository, https://zenodo.org/, accession number 7769978.

## Ethics statement

This study was approved by the Clinical Trials and Biomedical Ethics Committee of Sichuan University. All participants provided written informed consent.

## Author contributions

QG, JL, WX, and WK were designed for current research. HZX, YH, WL, JL, WK, XY, and HFX performed the acquisition of data. HFX and TL analyzed the data and wrote the manuscript. All authors contributed to the discussion of the results and have approved the final manuscript to be published.

## Funding

This study was supported by the National Natural Science Foundation of China (ZYJC2102981621003 and 2022YFC2009 900/2022YFC2009906), 1.3.5 project for disciplines of excellence, West China Hospital, Sichuan University (ZYJC21029) and National Clinical Research Center for Geriatrics, West China Hospital, Sichuan University (Z20191006). We would like to thank Editage (www.editage.cn) for English language editing. Regional Cooperation Project (Sichuan-Guangdong) of Sichuan Science and Technology Programs (2022YFQ0100).

## Conflict of interest

The authors declare that the research was conducted in the absence of any commercial or financial relationships that could be construed as a potential conflict of interest.

## Publisher’s note

All claims expressed in this article are solely those of the authors and do not necessarily represent those of their affiliated organizations, or those of the publisher, the editors and the reviewers. Any product that may be evaluated in this article, or claim that may be made by its manufacturer, is not guaranteed or endorsed by the publisher.

## Footnotes

^1^https://metascape.org

^2^https://string-db.org/

^3^http://www.cytoscape.org/
