# Supplementary material for: Genetic variations in the retrograde endocannabinoid signaling pathway in Chinese patients with major depressive disorder
Source: Front Neurol. 2023 Apr 20;14:1153509. doi: 10.3389/fneur.2023.1153509 (PMC10165312; doi:10.3389/fneur.2023.1153509)
Supplement: Supplementary file 1 [file Presentation_1.pdf]

Supplementary material

1 Whole-genome sequencing and data analysis

1.1 DNA quantification and library construction

Agarose gel electrophoresis (AGE) was used to analyze the degree of DNA degradation and the presence of RNA or protein contamination. Qubit was used for precise quantification of DNA concentrations. DNA samples with a content greater than 0.6 μg were used to build a library. For each sample, genomic DNA extracted from peripheral blood was fragmented to an average size of 180 to 280 bp and used to create a DNA library based on Illumina paired-end protocols (Illumina Inc., San Diego, CA, USA). The Agilent SureSelect Human All ExonV6 Kit (Agilent Technologies, Santa Clara, CA, USA) was used for exome capture, in accordance with the manufacturer's instructions. For genomic DNA sequencing, the Illumina Novaseq 6000 platform (Illumina Inc., San Diego, CA, USA) was used to generate 150-bp paired-end reads with a minimum coverage of 100× for ~99% of the genome (mean coverage of 100×). After sequencing, base-call file conversion and demultiplexing were performed using bcl2fastq software (Illumina). The resulting fastq data were analyzed using in-house quality control software to remove low-quality reads.

1.2 Whole-exome sequencing (WES) and annotation

Reads were aligned to the reference human genome (hg38) using the Burrows-Wheeler Aligner (BWA) (Li and Durbin, 2009), and duplicate reads were marked using Sambamba tools (Tarasov et al., 2015). Single nucleotide variants (SNVs) and indels were called using the Genome Analysis Toolkit (GATK 3.7) (DePristo et al., 2011) to generate a gVCF file. Annotation was performed using ANNOVAR (Wang et al., 2010). Low-frequency variants and rare mutations were filtered (MAF < 0.05). Minor allele frequencies were obtained from public datasets, including the 1,000 Genomes Project (1KGP) (Auton et al., 2015), esp6500siv2_all (http://evs.gs.washington.edu/EVS), the Exome Aggregation Consortium (ExAC), and the Genome Aggregation Database (gnomAD data) (Lek et al., 2016).

1.3 Filtration

GTX.Digest.VCF (Jiang et al., 2019) was used to further filter the candidate pathogenic genes. The system uses variant calling data (gVCF files generated via GATK) and phenotype information as the input. We used this system to set the filtration conditions related to the genotypes and phenotypes. GTX.Digest.VCF then ranks the variants and genes using a trained neural network model. For gene annotation, we utilized Clinvar (Landrum et al., 2016), OMIM (Amberger et al., 2015), and the mutation disease knowledge base generated via text mining.

To better predict the harmfulness of variation, we first utilized the classification system of the American College of Medical Genetics and Genomics (ACMG), which classifies variations as pathogenic, likely pathogenic, of uncertain significance, likely benign, or benign (Richards et al., 2015). Variations were then screened according to their scores using the SIFT (Kumar et al., 2009), Polyphen (Adzhubei et al., 2010), MutationTaster (Schwarz et al., 2010), and CADD (Kircher et al., 2014) software programs. Potentially deleterious variations were retained if the scores from more than half of the four software programs supported their potential harmfulness (Muona et al., 2015).
